# miR-425-5p Inhibits Differentiation and Proliferation in Porcine Intramuscular Preadipocytes

**DOI:** 10.3390/ijms18102101

**Published:** 2017-10-06

**Authors:** Fen-Fen Chen, Yan Xiong, Ying Peng, Yun Gao, Jin Qin, Gui-Yan Chu, Wei-Jun Pang, Gong-She Yang

**Affiliations:** 1 Laboratory of Animal Fat Deposition and Muscle Development, College of Animal Science and Technology, Northwest A&F University, Yangling 712100, China; ffchen03@sina.com (F.-F.C.); xiongyan0910@126.com (Y.X.); py9101@163.com (Y.P.); gy113@nwsuaf.edu.cn (Y.G.); qinjin19921026@sina.com (J.Q.); yz97721@gmail.com (G.-Y.C.); pwj1226@nwsuaf.edu.cn (W.-J.P.); 2 Faculty of Life Sciences, Southwest Forestry University, Kunming 650224, China

**Keywords:** miR-425-5p, porcine intramuscular preadipocytes, proliferation, KLF13, differentiation, PPARγ signaling

## Abstract

Intramuscular fat (IMF) content affects the tenderness, juiciness, and flavor of pork. An increasing number of studies are focusing on the functions of microRNAs (miRs) during porcine intramuscular preadipocyte development. Previous studies have proved that miR-425-5p was enriched in porcine skeletal muscles and played important roles in multiple physiological processes; however, its functions during intramuscular adipogenesis remain unclear. To explore the role of miR-425-5p in porcine intramuscular adipogenesis, miR-425-5p agomir and inhibitor were used to perform miR-425-5p overexpression and knockdown in intramuscular preadipocytes, respectively. Our results showed that the agomir of miR-425-5p dramatically inhibited intramuscular adipogenic differentiation and downregulated the expression levels of adipogenic marker genes PPARγ, FABP4, and FASN, whereas its inhibitor promoted adipogenesis. Interestingly, the agomir repressed proliferation of porcine intramuscular preadipocytes by downregulation of cyclin B and cyclin E. Furthermore, we demonstrated that miR-425-5p inhibited adipogenesis via targeting and repressing the translation of KLF13. Taken together, our findings identified that miR-425-5p is a novel inhibitor of porcine intramuscular adipogenesis possibly through targeting *KLF13* and subsequently downregulating *PPARγ*.

## 1. Introduction

Intramuscular fat (IMF) content is critical for various meat quality parameters such as muscle color, firmness, and water-holding capacity [1]. Previous studies have provided the methods for isolation and in vitro culture of primary intramuscular preadipocytes, which facilitates the understanding of mechanisms of IMF deposition [2,3]. Excess adipose tissue can be the consequence of both increased fat-cell number and increased fat-cell size [4,5]. The number of intramuscular adipocytes present in an organism is largely determined by the intramuscular preadipocyte differentiation process [1,6,7]. Thus, further understanding the differentiation of pig intramuscular preadipocytes may provide valuable information in animal body development and improvement of meat quality.

MicroRNAs are a class of short and endogenously initiated non-coding RNAs that can post-transcriptionally control gene expression via either translational repression or mRNA degradation [8,9]. MiRNAs have been found to be involved in numerous physiological and pathological processes, including cell proliferation, differentiation, apoptosis, tumorigenesis, and neuronal development [10]. Recently, many studies have indicated that miRNAs play key roles in regulating adipogenesis [11]. In porcine adipogenesis, miR-103, miR-181a, miR-15a/b, miR-196a, miR-17, miR-21, miR-143, and miR-146a-5p promoted porcine preadipocyte differentiation [12,13,14,15,16,17], while miR-125a, miR-145, miR-191, miR-199a-5p, miR-375, and miR-429 impaired porcine preadipocyte differentiation [18,19,20,21,22,23].

Mature miR-425-5p was primarily found as a potential oncogene in cancer [24,25]. It has recently been reported to regulate cell proliferation, cell cycle, apoptosis and metastasis, immunology, and inflammation [24,25,26,27,28,29]. Recently, miR-425-5p was shown to be differently regulated during tumorigenesis and to be involved in regulating key pathways such as PI3K/AKT signaling, T cell receptor and VEGF/p38 signaling, or some of the genes which participate these processes [25,27,28,30]. In these biological processes, miR-425-5p functioned by targeting different mRNAs. The published targeting genes of miR-425-5p, such as insulin-like growth factor 1 (IGF-1) and mothers against decapentaplegic homolog 2 (SMAD family member 2, SMAD2), played roles in adipocyte differentiation, [25,30]. Here, we identified that miR-425-5p was relatively highly expressed in porcine muscle and subcutaneous adipose tissue. However, little is known about the expression and functions of miR-425-5p in porcine intramuscular adipogenesis.

In this study, we monitored the expression pattern of miR-425-5p during porcine intramuscular preadipocyte adipogenesis. Overexpression of miR-425-5p attenuates the lipid accumulation during porcine intramuscular preadipocyte differentiation, which is consistent with the block of peroxisome proliferator activated receptor γ (PPARγ) signaling. Bioinformatic prediction and further experimental validation showed that Krüppel-like transcription factor 13 (KLF13) is a target of miR-425-5p in porcine adipocytes. Furthermore, we used flow cytometry, Western blot, and CCK-8 assay to show that the overexpression of miR-425-5p levels leads to inhibiting the proliferation of porcine intramuscular preadipocytes. Taken together, our study suggests that miR-425-5p is a negative regulator for porcine intramuscular adipogenesis. Our data provides new insights in pork quality improvement.

## 2. Results

### 2.1. Expression Profiles of miR-425-5p in Porcine Tissues and during Intramuscular Preadipocytes Adipogenesis

**Figure 1:**
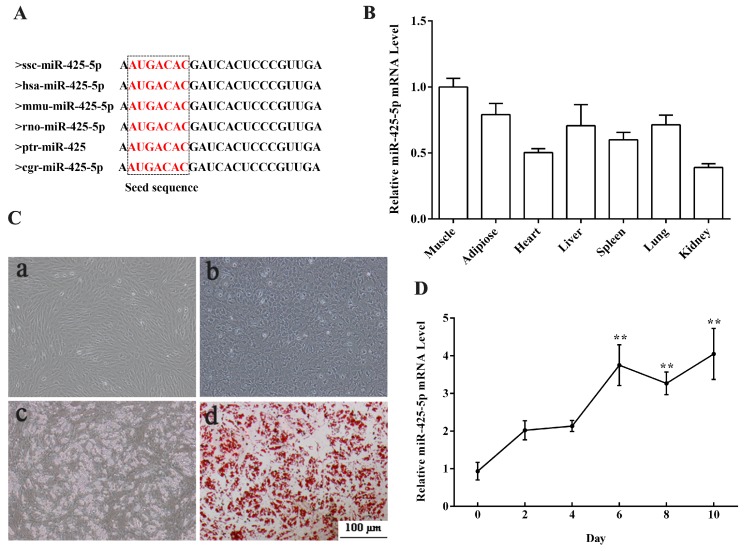
Expression profile of miR-425-5p in different porcine tissues and during porcine intramuscular preadipocyte differentiation. (**A**) Mature miR-425-5p sequence was conserved among species; (**B**) expression of miR-425-5p was analyzed by real-time PCR in seven different tissues of 180-day old Guanzhong Black pigs; (**C**) photomicrographs showing cell morphology change during porcine intramuscular preadipocyte differentiation ((**a**) porcine intramuscular preadipocyte before 3-isobutyl-1-methylxanthine (IBMX)–Dexamethasone (DEX)–insulin (DMI) induction; (**b**) porcine intramuscular preadipocyte 2 days after DMI induction; (**c**) porcine intramuscular preadipocyte 8 days after DMI induction; (**d**) porcine intramuscular preadipocyte 8 days after DMI induction staining with Oil Red O); (**D**) expression of miR-425-5p during porcine intramuscular preadipocyte differentiation and the expression at the day 0 as a control. U6 small nuclear RNA was used as a reference gene. Results were presented as means ± SEM, *n* = 3; ** *p* < 0.01.

Mature miR-425-5p sequence was highly conserved in virous mammals including pig, human, mouse, and rat (Figure 1A). To determine the tissue expression profile of miR-425-5p, total RNAs from seven tissues of adult Guanzhong Black piglets were extracted and real-time PCR (RT-PCR) was performed. Results showed (Figure 1B) that miR-425-5p was highly expressed in muscle (longissimus dorsi) and subcutaneous adipose tissue. To study the role of miR-425-5p in porcine intramuscular adipogenesis, porcine intramuscular preadipocytes were isolated. After 8 days induction, intramuscular adipocytes were fully differentiated and fulfilled with large lipid droplets (Figure 1C). Then RNA was extracted from adipogenic cells at 0, 2, 4, 6, 8, and 10 days of differentiation to detect the miR-425-5p expression pattern during intramuscular adipocyte differentiation. Our data revealed that miR-425-5p transiently increased during intramuscular adipocyte adipogenesis (Figure 1D). Taken together, these data suggested that miR-425-5p might be associated with intramuscular adipocytes adipogenesis.

### 2.2. miR-425-5p Inhibits Differentiation of Porcine Intramuscular Preadipocytes

**Figure 2:**
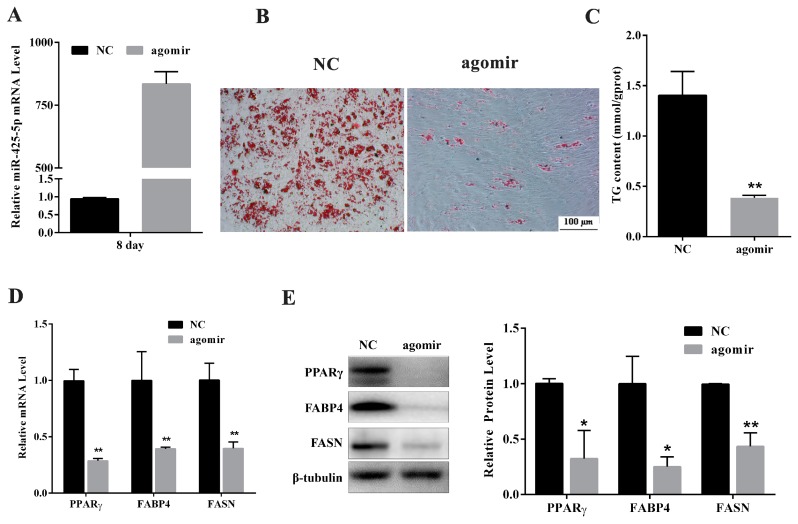
Overexpression of miR-425-5p inhibits porcine intramuscular adipogenesis. (**A**) miR-425-5p overexpression efficiency was detected by RT-qPCR; (**B**) the morphological changes and lipid accumulation of porcine differentiated intramuscular adipocytes observed by Oil Red O staining; (**C**) triglyceride (TG) content assay of differentiated adipocytes; (**D**) the relative mRNA level of adipocyte specific genes including *PPARγ*, *FABP4*, and *FASN*; (**E**) protein level of PPARγ, FABP*4*, and FASN genes were examined by Western blotting, β-tubulin was used as a loading control. All the cells were measure at day 8 of adipogenic differentiation. Results were presented as means ± SEM, *n* = 3; * *p* < 0.05; ** *p* < 0.01.

To identify the potential function of miR-425-5p for intramuscular adipogenesis, miR-425-5p agomir or negative control (NC) was transfected to intramuscular preadipocytes. RT-qPCR assay showed that miR-425-5p overexpressed 800-fold at the eighth day of differentiation after transfected with the agomir (Figure 2A). Gain function of miR-425-5p expression inhibited intramuscular preadipocyte differentiation indicated by reduction of both Oil Red O staining signal (Figure 2B) and triglyceride (TG) contents at day 8 of adipogenic induction (*p* < 0.01) (Figure 2C). The mRNA levels of adipogenic markers, *PPARγ* (*p* < 0.01), *FABP4* (*p* < 0.01), and *FASN* (*p* < 0.01), were significantly suppressed in miR-425-5p agomir treated cells compared with those of NC (Figure 2D). Consistently, the protein levels of PPARγ, adipocyte fatty acid-binding protein 4 (FABP4), and fatty acid synthase (FASN) all decreased in the miR-425-5p overexpression group at day 8 of differentiation (Figure 2E).

**Figure 3:**
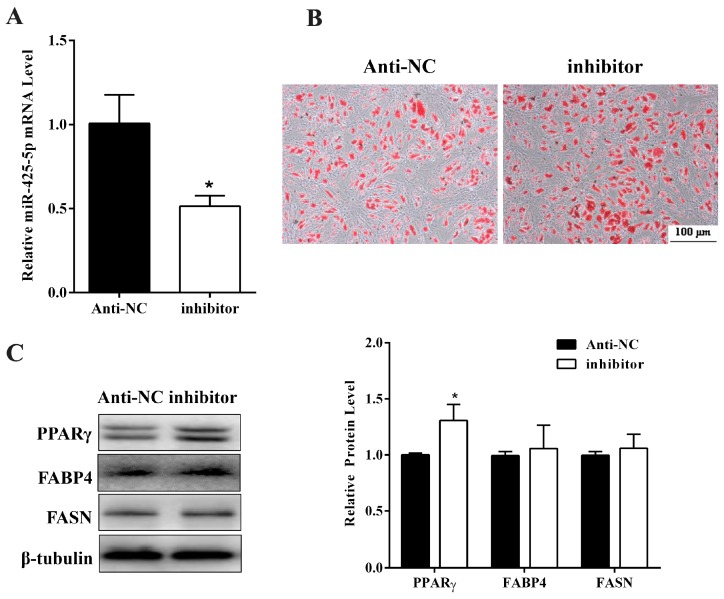
Inhibition of miR-425-5p promoted porcine intramuscular adipocyte differentiation. (**A**) The miR-425-5p expression was measured by RT-qPCR at day 8 after differentiation; (**B**) Oil Red O staining of intramuscular adipocytes showed antagonism of miR-425-5p increased the lipid accumulation; (**C**) protein expression of PPARγ, FABP4, and FASN were determined by Western blotting, β-tubulin was used as a loading control. Results were presented as means ± SEM, *n* = 3; * *p* < 0.05.

Next, the anti-sense oligo nucleotides (inhibitor) targeting miR-425-5p or NC was transfected to porcine primary intramuscular adipocyte (the same method used for agomir transfection) to confirm the role of miR-425-5p in adipocyte differentiation. RT-qPCR showed that miR-425-5p was effectively inhibited (Figure 3A). As expected, Oil Red O staining (Figure 3B) showed an increased neutral lipid accumulation in inhibitor transfected cells. Although protein levels of FABP4 and FASN showed no difference (*p* > 0.05) (Figure 3C), this level of PPARγ increased significantly at day 8 of differentiation in treatment cells compared with NC (*p* < 0.05) (Figure 3C). Taken together, these data indicated that miR-425-5p inhibited porcine intramuscular preadipocyte differentiation.

### 2.3. miR-425-5p Can Target KLF13 in Porcine Intramuscular Preadipocytes

**Figure 4:**
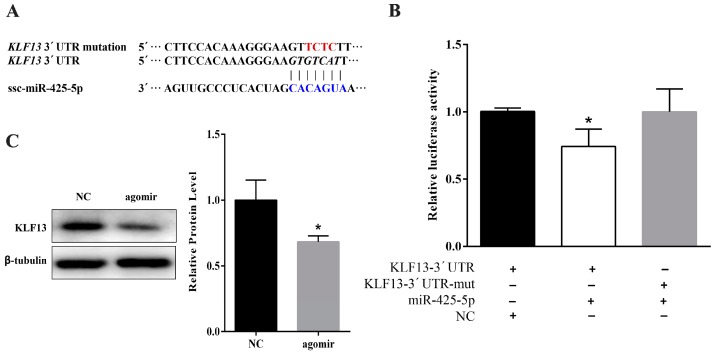
MiR-425-5p targets 3′UTR of KLF13. (**A**) Target site of miR-425-5p within porcine KLF13 mRNA 3′UTR and the mutation design of its 3′UTR; (**B**) the luciferase assay of MiR-425-5p targeting 3′UTR of KLF13. psiCHECK™-2 Vectors, containing either the KLF13 3′UTR or the KLF13 3′UTR with a mutation in the miR-425-5p seed region, were transfected into HEK293 cells either alone or in combination with negative control (NC) or miR-425-5p agomir. Renilla luciferase activity was normalized to firefly luciferase; (**C**) KLF13 protein level in transfection of miR-425-5p agomir cells was detected by Western blotting at day 8 of differentiation, β-tubulin was used as a loading control. Results were presented as means ± SEM, *n* = 3; * *p* < 0.05.

To reveal the underlying mechanism of miR-425-5p in porcine intramuscular preadipocyte differentiation, bioinformatic prediction software was used and found that KLF13 was a candidate target gene (Figure 4A). Therefore, the 3’UTR containing miR-425-5p targeted sites was cloned and inserted downstream of luciferase gene in the psiCHECKTM-2 reporter plasmid. Moreover, the mutated plasmid was constructed by inserting KLF13 3′UTR with mutated miR-425-5p binding site. The wild-type (psiCHECK-KLF13-UTR) or mutated (psiCHECK-KLF13-muta) plasmid was co-transfected with the miR-425-5p agomir into 293T cells, respectively. After 48 h of transfection, the luciferase activity of the miR-425-5p group was significantly lower than that of the NC group (*p* < 0.05), and this reduction disappeared in the mutation group (Figure 4B). Further, protein levels of KLF13 were significantly decreased by miR-425-5p overexpression in porcine intramuscular adipocytes (Figure 4C). Altogether, KLF13 was a bona fide target of miR-425-5p.

A previous study proved that KLF13 is a key pro-adipogenic factor through regulating PPARγ transactivation during porcine adipocyte differentiation [31]. In the present study, miR-425-5p is a translational repressor of its KLF13 target gene during cell differentiation (Figure 4A,B). Overexpression of miR-425-5p in intramuscular adipocyte dramatically reduced KLF13 (*p* < 0.05) protein expression (Figure 4C), which is associated with inhibition of the expression of PPARγ (*p* < 0.05) (Figure 2E). In addition, knockdown of miR-425-5p promoted the protein level of PPARγ (Figure 3C). Therefore, we speculated that miR-425 may indirectly regulate the expression of PPARγ through the targeting of KLF13.

### 2.4. miR-425-5p Inhibits Porcine Intramuscular Preadipocytes Proliferation

**Figure 5:**
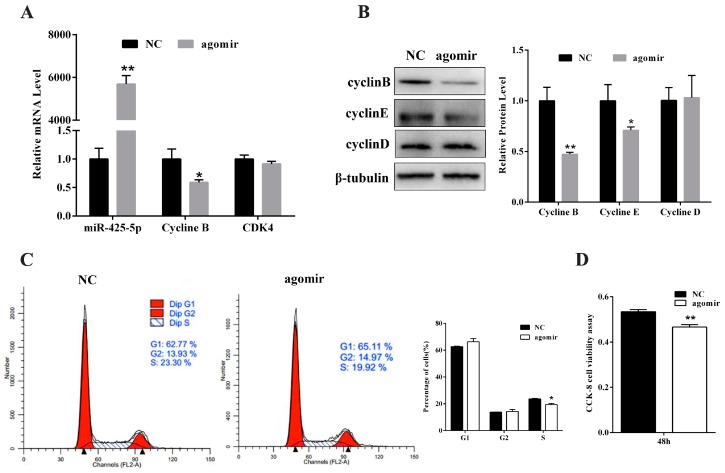
MiR-425-5p inhibits porcine intramuscular preadipocyte proliferation. MiR-425-5p agomir or negative control (NC) were transfected into intramuscular preadipocyte when cells reached 40% confluence and cells were harvested after 48 h of transfection. (**A**) RNA levels of miR-425-5p, Cyclin B, and CDK4 were detected by RT-qPCR; (**B**) the protein level of cell cycle genes, Cyclin B, Cyclin E, and Cyclin D, were analyzed by Western blotting; (**C**) cell-cycle analysis of intramuscular preadipocyte using flow cytometry; (**D**) cell proliferation was accessed by CCK-8 assay. Results were presented as mean ± SEM, *n* = 3; * *p* < 0.05. ** *p* < 0.01.

To elucidate whether miR-425-5p may also function in proliferating intramuscular preadipocytes, miR-425-5p agomir or NC was transfected to intramuscular preadipocytes at 40% density. After 48 h, we analyzed the expression of miR-425-5p by RT-PCR and results showed miR-425-5p with a 6000-fold increase compared with the NC (Figure 5A). The mRNA expression levels of Cyclin B, a positive regulator of cell proliferation, was downregulated (*p* < 0.05) upon overexpression of miR-425-5p. In addition, levels of CDK4 showed no difference (*p* > 0.05) (Figure 5A). Meanwhile, compared to control, the protein levels of Cyclin B (*p* < 0.01) and Cyclin E (*p* < 0.05) were downregulated (*p* < 0.05), and levels of Cyclin D showed no difference (*p* > 0.05) (Figure 5B). After 48 h of transfection, we also carried out flow cytometry to detect the alterations in the cell cycle caused by miR-425-5p overexpression. The data showed that overexpressed miR-425-5p decreased the number of S-phase intramuscular preadipocytes (Figure 5C). CCK-8 assay confirmed that miR-425-5p overexpression led to decreased cell number at 48 h post-transfection compared to the NC group (Figure 5D). Collectively, these data indicated that miR-425-5p repressed the proliferation of porcine intramuscular preadipocytes.

## 3. Discussion

Mature miR-425-5p is highly conserved in mammals, which suggests that miR-425-5p may be important for gene regulation and is thus preserved during evolution. In this study, we aimed to investigate the physiological role of miR-425-5p associated with adipogenesis in porcine intramuscular preadipocytes. We found that miR-425-5p level was increased during intramuscular preadipocyte differentiation, indicating that miR-425-5p might regulate intramuscular preadipocyte adipogenesis. We showed that the expression levels of PPARγ, FASN, and FABP4 declined in cells transfected with miR-425-5p agomir. Consistently, blocking of miR-425-5p expression enhanced intramuscular preadipocyte differentiation. These data suggest that miR-425-5p is a negative regulator for intramuscular preadipocyte adipogenesis.

Krüppel-like factors (KLFs) are important regulators of cell differentiation, proliferation, and a number of other cellular processes [32]. In recent years, a total of 10 KLF proteins (KLF2-9, KLF13, and KLF15) have been proved to either promote or inhibit adipocyte differentiation [22,31]. Specifically, KLF13 has been proved to be a key pro-adipogenic factor through regulating PPARγ transactivation at the early stage of porcine adipocyte differentiation [31]. In this study, we showed that miR-425-5p could target KLF13, which is associated with impairing the protein expression of PPAR*γ.* This is further supported by the downregulated level of its downstream genes, including FABP4 and FASN [33].

Previous studies have reported that miR-425-5p displayed inconsistent effects on the proliferation of different cell types. In melanoma cells, enhancing expression of miR-425 inhibited proliferation by targeting IGF-1, which plays important roles in the PI3K/AKT signaling pathway [30]. Other studies have indicated that miR-425-5p enhanced cell proliferation via suppression of Phosphatase and tensin homolog (PTEN), SMAD2, catenin alpha 3 (CTNNA3), and cerebral cavernous malformations 3 (CCM3) expression [25,26,29,34]. The discrepancy of the function of miR-425-5p in different cell lines may be due to the fact that miRNA-mRNA targeting and the interplay relationship differs among tissue and cell types. Our flow cytometry and Western blot assay showed that miR-425-5p impaired the expression of cell cycle genes in intramuscular preadipocytes and significantly slowed down the cell cycle progression of these cells. For the preadipocyte adipogenic differentiation process, clonal expansion is critical for terminal adipogenic differentiation. This suggested that miR-425-5p could also restrain adipogenic differentiation by negatively regulating clonal expansion [35,36].

In conclusion, our data indicated that miR-425-5p is a negative regulator of porcine intramuscular preadipocyte proliferation and differentiation by targeting KLF13. These findings provide new molecular insights into improving meat quality in the practice of animal husbandry.

## 4. Materials and Methods

### 4.1. Animals

Six Guanzhong Black piglets at 3 days were provided by the Experimental Farm of Northwest A&F University (Yangling, China). All piglets were sacrificed by CO_2_ asphyxiation. The procedure was conducted in accordance with the Institutional Animal Care and Use Committee of Northwest A&F University (14-233, 10 December 2014).

### 4.2. Porcine Intramuscular Preadipocytess Isolation and Induction

Longissimus dorsi muscles were aseptically isolated and all visible connective tissue was removed. The isolated muscle tissues were washed three times in pH 7.4 phosphate buffered saline (PBS) with 200 U/mL penicillin–streptomycin. The tissue was then minced to 2–3 mm in serum free dulbecco’s modified eagle medium/F12(DMEM/F12) (Gibco BRL Co., LTD, San Francisco, CA, USA) medium with 2 mg/mL type-II collagenase (Invitrogen, Carlsbad, CA, USA), and 150 mg/mL bovine serum albumin (Sigma, St. Louis, MO, USA). The finely minced tissues were digested at 37 °C for 120 min in a shaking water bath with 40 rpm speeds. The solution added equal volume DMEM/F12 growth medium supplemented with 10% fetal bovine serum (FBS, Hyclone, Thermo scientific, Waltham, MA, USA) and 100 IU/mL penicillin–streptomycin. The digest was passed through sterile 178 and 74 mm steel mesh filters to isolate digested cells. Cells were rinsed with serum free DMEM/F12 medium (FBS free) and centrifuged twice at 1500× *g* for 10 min, and then resuspended in DMEM/F12 medium. Viable cells were counted using 0.4% trypan blue and a Countstar automatic cell counter (Inno-Alliance Biotech, Wilmington, DE, USA). Cells were resuspended in DMEM/F12 and plated at a density 6 × 10^5^ per 60-mm culture dish, and cultured in a 5% CO_2_ incubator at 37 °C. Because preadipocytes attach much earlier than myoblasts, the cultures cells were rinsed with PBS three times 1 h after plating to remove insoluble myofibrillar proteins and other insoluble debris [2,22,37]. Cells were cultured in growth medium until they reached 80% confluence and digested with 0.05% trypsin, which contained 0.5 mmol/L EDTA, collected by centrifugation 1000× *g* for 5 min, then resuspended in growth medium and plated at a density of 5 × 10^4^ cells/cm in 6-well plate and used to induce differentiation for research. Briefly, when the cells reached confluence, the medium was changed with induction medium, which is the DMEM/F12 supplement with 10% FBS, 100 U/mL penicillin–streptomycin, 0.5 mM IBMX, 1 nM DEX, and 5 ng/mL insulin (IBMX, DEX and insulin were purchased from Sigma). After 2 days, the medium was changed with differentiation medium, including DMEM/F12 supplement with 10% FBS, 5 ng/mL insulin, and 100 U/mL penicillin–streptomycin, then changed every 2 days until day 8. At this stage, the cells were differentiated and used for subsequent experiments.

### 4.3. Transfection of miRNA Agomir and Inhibitor

Porcine intramuscular preadipocytes were seeded in 12-well or 6-well plates, and 50 nM miR-425-5p agomir or negative control (NC) (Genepharma, Shanghai, China) was transfected into cells of 40% density using X-tremeGENE siRNA Transfection Reagent (No.04476115001, Roche Diagnostics GmbH, Mannheim, Germany) and Opti-MEM (Gibco BRL Co., LTD) culture medium according to the manufacturers’ protocol. After 48 h, the culture medium was changed to fresh medium in order to study the proliferation of intramuscular preadipocytes. Cells were harvested 48 h after transfection. When transfected with miR-425-5p inhibitor, the protocol was the same as with agomir, but the final concentration of miR-425-5p inhibitor was 100 nM, according to the introductions. Nevertheless, for adipogenic differentiation, cells were transfected when density of intramuscular preadipocytes reached 70%. When cells grew to confluence after transfection, adipogenic differentiation was initiated by switching to differentiation medium.

### 4.4. RNA Extraction and RT-PCR

Total RNA was extracted from the cells using TRIzol reagent (Takara, Kyoto, Japan). cDNA was synthesized and RT-qPCR reactions were performed in triplicate using the SYBR green kit (Takara) with a Bio-Rad iQ™5 system. microRNA assay was detected with stem-loop primers purchased from RiboBio. U6 small nucleolar RNA (Guangzhou RiboBio Co., LTD, Guangzhou, China) was used for the normalization. The primer sequences used for genes studied are listed in Table 1. The final relative expression fold differences, with gene expression NC as a control, were calculated as 2^−ΔΔ*C*t^ for each gene.

### 4.5. Western Blotting

As previously described [22], cells were washed twice with PBS and solubilized with radio immunoprecipitation assay (RIPA) buffer (Vazyme Biotec Co., LTD, Nanjing, China) supplemented with protease inhibitor (Sigma). Lysates were quantitated with bicinchoninic acid (BCA) kit (Vazyme Biotec Co., LTD); equivalent amounts of protein were subjected to electrophoresis, and transferred onto PVDF membranes (Millipore, Billerica, MA, USA). Blocking with 5% dried skimmed milk in TBST (Tris Buffered Saline and 0.1% Tween 20) for 2 h at room temperature, the membranes were incubated with different primary antibodies, including KLF13 (#41724, SAB, Maryland, MD, USA), FASN (sc-20140 AC, Santa Cruz Biotechnology, Inc., Santa Cruz, CA, USA), FABP4 (sc-18661, Santa Cruz Biotechnology, Inc.), PPARγ (#2435, CST, Cell Signaling Technology, Inc., Danvers, MA, USA), and β-tubulin (KM9003, Sungene, Tianjin, China), at 4 °C overnight. After washing, the membrane was incubated with HRP-conjugated secondary antibodies for 1 h at room temperature and developed with ChemiDoc^TM^ XRS + Chem iluminescence detection system (Bio-Rad, Hercules, CA, USA). Image Lab5.2 was used for densitometric analysis of the expressed protein bands.

### 4.6. Oil Red O Staining

The extent of differentiation was determined by the amount of lipid accumulation by Oil Red O (ORO) staining, as described previously [38]. Cells were observed and photographed by TE2000-S microscope (Nikon, Tokyo, Japan).

### 4.7. Measurement TG

TG contents were detected on day 8 of differentiation. Cells were digested by 0.25% trypsin and then homogenized by ultrasonic cracking (Sonics Vcx105, Newtown, CT, USA). Lysates were measured using TG test kit (Nanjing Jiancheng Bioengineering Institute, Nanjing, China). TG content analysis was conducted according to the manufacturer’s protocol.

### 4.8. Luciferase Reporter Assay

The 3′-UTRs of porcine KLF13 containing miR-425-5p targeted sites were cloned from porcine adipocytes cDNA using primers tagged with XhoI and NotI (Takar) cutting sites. The wild-type or mutated 3′-UTR fragment was cloned into psiCHECKTM-2 Vector (Promega, Madison, WI, USA) at the 3′-end of the Renilla gene. The structured 3′-UTR dual-luciferase vectors/the 3′-UTR point mutations at positions 2–6 of the KLF13 seed region dual-luciferase vectors and miR-425-5p agomir/NC were co-transfected into porcine intramuscular preadipocyte using X-tremeGENE HP DNA Transfection Reagent. Cells were harvested at 48 h post-transfection and assayed based on the manufacturer’s instructions (Promega).

### 4.9. Flow Cytometry

Porcine intramuscular preadipocytes being the stable phase of cell growth, cells were seeded in 6-well culture plates at a density of 1.6 × 10^6^ cells per well. After 24 h, cells were transfected with miR-425-5p agomir/NC by X-tremeGENE HP siRNA Transfection Reagent (Roche), and cells were washed three times with PBS, harvested at 48 h post-transfection, and then stained with DNA staining solution (CCS01; Multi Sciences, Hangzhou, China), and left to incubate for 30 min. Finally, a flow cytometry instrument (Becton Dickinson, Franklin Lakes, NJ, USA) was used to analyze the proliferation phase of the cells.

### 4.10. Cell Vitality Analysis

Porcine intramuscular preadipocytes were seeded in 96-well plates in the growth medium with a density of 2 × 10^3^ cells per well. After transfection of agomir/NC for 24 h, the medium was removed and 90 μL or fresh medium mix with 10 μL CCK-8 reagents (Vazyme Biotec Co., LTD) were added to each well. After incubation for 2 h at 37 °C, the 96-well culture plates were agitated for 1 min and the absorbance at 450 nm for each well was detected using a PerkinElmer VICTOR X5 (PerkinElmer lnc, Waltham, MA, USA).

### 4.11. Statistical Analysis

All experiments were carried out at least three times. GraphPad Prism 6.0 was utilized to graph the results. Data were analyzed using SPSS 17.0 software (SPSS science, Chicago, IL, USA) and evaluated for differences between groups by unpaired Student’s *t*-test. Data are presented as means ± SEM. A value of *p* < 0.05 (*) was considered to be statistically significant.

## Figures and Tables

**Table 1:** Primer sequences used in this study.

Gene	Accession Number	Orientation	Primer Sequences (5′-3′)	Production Length (bp)
PPARγ	NM_214379	Forward	AGGACTACCAAAGTGCCATCAAA	142
		Reverse	GAGGCTTTATCCCCACAGACAC	
FABP4	HM_453202	Forward	GAGCACCATAACCTTAGATGGA	121
		Reverse	AAATTCTGGTAGCCGTGACA	
FASN	EF589048.1	Forward	AGCCTAACTCCTCGCTGCAAT	196
		Reverse	TCCTTGGAACCGTCTGTGTTC	
Cyclin B	NM_001170768	Forward	AATCCCTTCTTGTGGTTA	104
		Reverse	CTTAGATGTGGCATACTTG	
CDK4	NM_001123097	Forward	ATCAGCACGGTTCGTGAAGT	133
		Reverse	GCTCAAACACCAGGGTCACT	
GAPDH	KJ786424	Forward	AGGTCGGAGTGAACGGATTTG	118
		Reverse	ACCATGTAGTGGAGGTCAATGAAG	

## Abbreviations

PPARγ	Peroxisome proliferator activated receptor γ
FABP4	Adipocyte fatty acid-binding protein 4
FASN	Fatty acid synthase
KLF13	Krüppel-like transcription factor 13
Cyclin E	Cell cycle protein E
Cyclin B	Cell cycle protein B
Cyclin D	Cell cycle protein D
CDK4	Cyclin-dependent kinases 4
ssc	Susscrofa
hsa	Homo sapiens
mmu	Musmusculus
rno	Rattusnorvegicus
ptr	Pan troglodytes
cgr	Cricetulusgriseus
